# N-myc and STAT interactor promotes poly(I:C)-induced pulmonary coagulopathy via STAT3-dependent tissue factor

**DOI:** 10.3389/fimmu.2026.1873798

**Published:** 2026-07-02

**Authors:** Jihua Zhang, Yongqiang Zhou, Qingying Mu, Xiufen Zheng, Jing Qin, Huanhuan Liang

**Affiliations:** 1 School of Pharmaceutical Sciences, Sun Yat-sen University Shenzhen Campus, Shenzhen, China; 2 Shenzhen Key Laboratory for Systems Medicine in Inflammatory Diseases, School of Medicine, Sun Yat-sen University, Shenzhen, China

**Keywords:** coagulopathy, damage-associated molecular pattern, N-myc and STAT interactor, STAT3 signaling, tissue factor, viral pneumonia

## Abstract

**Background:**

Viral pneumonia-associated coagulopathy is a major determinant of mortality, yet the upstream molecular drivers initiating the coagulation cascade remain incompletely understood.

**Methods:**

We integrated bulk and single-cell transcriptomics to map viral infection signatures. Mechanistic and therapeutic evaluations were performed in a poly(I:C)-induced pneumonia model utilizing candidate gene-knockout mice and STAT3 inhibition (Stattic), with endpoints including lung injury, tissue factor (TF) expression, and microthrombosis.

**Results:**

We identified N-myc and STAT interactor (NMI) as a critical damage-associated molecular pattern (DAMP) promoting poly(I:C)-induced pulmonary coagulopathy. Transcriptomic analyses across human and murine viral pneumonia datasets revealed robust NMI upregulation, which strongly correlated with coagulation markers. Mechanistically, extracellular NMI activates alveolar epithelial STAT3 signaling, directly upregulating TF to initiate the extrinsic coagulation cascade. *In vivo, Nmi* ablation or pharmacological STAT3 inhibition significantly attenuated lung microthrombosis and coagulopathy. Notably, this protective phenotype in *Nmi* knockout mice was reversed by recombinant NMI administration.

**Conclusion:**

Our findings elucidate the NMI-STAT3-TF axis as a key contributor to pulmonary coagulopathy. Targeting this axis may offer a potential preclinical therapeutic strategy for managing thrombotic complications in virus-induced lung inflammation.

## Introduction

Respiratory viral infections, including influenza and COVID-19, pose a persistent global public health threat, causing substantial morbidity and mortality (1, 2). While respiratory failure is a primary concern, these infections are frequently complicated by a dysregulated hemostatic response known as “immunothrombosis” (3), a physiological defense mechanism that, when uncontrolled, evolves into pathological coagulopathy (4, 5). Coagulopathy in coronavirus infection has been shown to be associated with high mortality (6, 7). In its most severe form, this manifests as disseminated intravascular coagulation (DIC), characterized by widespread microvascular thrombosis, consumption of clotting factors, and subsequent organ failure (8, 9). Although anticoagulant therapies have shown partial clinical benefits in hospitalized patients (10, 11), dysregulated coagulation remains strongly associated with poor outcomes in severe viral pneumonia. In an early COVID-19 cohort, 71.4% of non-survivors fulfilled the diagnostic criteria for DIC, underscoring the close association between uncontrolled coagulation and fatal disease progression (12). Therefore, deciphering the precise molecular mechanisms driving pathological coagulation activation is urgent for developing targeted therapeutic interventions.

The initiation of the extrinsic coagulation cascade, driven by the upregulation of tissue factor (TF), is the hallmark of viral-associated coagulopathy (13). Following viral entry, alveolar epithelial cells, the primary barrier of the lung, act as the first line of defense. Upon sensing viral RNA via pattern recognition receptors such as TLR3, these cells can undergo a phenotypic switch, upregulating TF expression and downregulating anticoagulant factors like thrombomodulin (14–16). While direct viral stimulation is a known trigger, the sustained and amplified coagulopathy seen in severe cases suggests the involvement of endogenous amplification loops. Damage-associated molecular patterns (DAMPs) released from necrotic or stressed cells, such as HMGB1 (high mobility group box-1 protein) (17), extracellular histones (18, 19), and S100A8/A9 (S100 Calcium Binding Protein A8/A9) (20), have been identified as potent contributors to TF-mediated thrombosis. However, distinct from these general necrosis markers, whether specific interferon-inducible proteins can actively drive pathological TF expression and initiate the coagulation cascade remains to be elucidated.

N-myc and STAT interactor (NMI), also known as an IFP35 (interferon induced protein 35) family protein, is an interferon-inducible protein recently characterized as a novel DAMP. Beyond its intracellular roles in interacting with STAT and MYC family proteins, extracellular NMI has been shown to amplify innate immune responses by activating macrophages and promoting the release of pro-inflammatory cytokines, including IL-6, TNF-α, and IL-1β (21–23). Notably, previous studies have demonstrated that genetic ablation of *Nmi* significantly attenuates lung inflammation and improves survival in PR8-induced influenza models (24), suggesting that NMI acts as a critical upstream mediator of viral pathogenesis. Despite these insights into its inflammatory role, whether NMI contributes to the thrombotic complications of viral pneumonia remains unknown.

Given the central role of TF in infection-associated coagulation, and the established association of NMI with the severity and prognosis of community-acquired pneumonia (CAP) and sepsis, we considered NMI a biologically plausible candidate linking viral sensing, inflammatory amplification, and pathological coagulation (25, 26). In this study, we integrated transcriptomic analyses of human patient cohorts and murine models to validate the robust upregulation of NMI. Furthermore, we dissected the specific intracellular signaling landscape driving pathological coagulation. We demonstrate that upon stimulation with the viral mimetic poly(I:C), activated alveolar epithelial cells release NMI, which may amplify TF-dependent coagulation through autocrine mechanisms via the STAT3 signaling axis. Our findings establish NMI as a key pathogenic factor promoting viral coagulopathy and highlight the potential of the NMI-STAT3 axis as a therapeutic target.

## Methods

### Data collection and processing

Publicly available transcriptomic datasets (GSE243217, GSE196350, GSE197868, GSE271812, and GSE292515) were downloaded from the Gene Expression Omnibus (GEO) database (https://www.ncbi.nlm.nih.gov/geo/). Specifically: GSE243217 contains peripheral blood RNA-seq data from 35 COVID-19 patients and 15 healthy individuals. GSE196350 comprises peripheral blood RNA-seq profiles from 51 influenza-infected patients and 18 healthy controls. GSE197868 includes lung tissue RNA-seq data from BALB/c mice infected with Respiratory Syncytial Virus (RSV) compared with uninfected controls (n=3 per group). GSE271812 provides lung tissue RNA-seq data from C57BL/6J mice infected with H1N1 influenza virus versus controls (n=4 per group). GSE292515 consists of single-cell RNA sequencing (scRNA-seq) profiling of lung tissues from young mice harvested at day 7 post-influenza infection.

### NMI expression and ROC assessment

NMI expression profiles were visualized using the “ggpubr” package. The discriminative performance of NMI for distinguishing COVID-19 and influenza from healthy controls was assessed by Receiver Operating Characteristic (ROC) analysis using the “pROC” package, with discriminative ability quantified by the Area Under the Curve (AUC).

### Functional enrichment analysis

To standardize gene identifiers, gene symbol-to-Entrez ID conversion was performed using the “org.Hs.eg.db” (for human datasets) and “org.Mm.eg.db” (for murine datasets) packages. Functional enrichment analyses, including Gene Ontology (GO) terms and Kyoto Encyclopedia of Genes and Genomes (KEGG) pathways, were conducted using the clusterProfiler package. Significant terms were identified based on an adjusted *P*-value < 0.05. The results were visualized using the ggplot2 package.

### ssGSEA and immune cell analysis

Single sample Gene Set Enrichment Analysis (ssGSEA) was performed using the “GSVA” package. Immune cell abundance and immune pathway activity were calculated from sample gene expression. Correlations between NMI expression and immune cells or pathways were calculated using Pearson correlation and visualized with “ggplot2”. Differences between control and influenza-infected groups were visualized as bar plots.

### Protein–protein interaction network analysis

Protein-protein interaction (PPI) network was constructed using the STRING database (https://string-db.org/). Interactions were filtered with a minimum required interaction score of 0.400. The resulting interaction data were then imported into Cytoscape (v3.10.4) for network visualization and layout optimization.

### scRNA-seq preprocessing, clustering, and cell type annotation

Single-cell RNA-seq data were processed using the “Seurat” package. Quality control was performed to filter low-quality cells. Cells were retained if they satisfied the following criteria: 300 < nFeature_RNA < 7000, mitochondrial gene percentage < 20%, and ribosomal gene percentage < 20%. Data were normalized using the NormalizeData function. Highly variable genes were identified using FindVariableFeatures, and data were scaled using ScaleData. Principal component analysis (PCA) was applied for dimensionality reduction, and batch effects were corrected using “Harmony”. Clustering was performed with the FindNeighbors and FindClusters functions, and the results were visualized using t-SNE.

Cell type annotation was conducted with CellMarker 2.0 database and further validated using canonical marker genes. In total, 17,276 cells were classified into 11 major cell types, including fibroblasts, macrophages, monocytes, epithelial cells (including alveolar type I/II), endothelial cells, T cells, NK cells, B cells, dendritic cells, ciliated cells and neutrophils.

### Differential analysis of scRNA-seq data

Cluster-specific marker genes for alveolar epithelial cells were identified using the FindAllMarkers function in “Seurat”, utilizing the Wilcoxon rank-sum test. To elucidate the functional heterogeneity driven by NMI, all alveolar epithelial cells (including those with zero *Nmi* expression) were stratified into *Nmi*-high and *Nmi*-low subpopulations based on the median expression level of *Nmi*. Differentially expressed genes (DEGs) between these two groups were identified (thresholds: |log2FC| > 0.25 and adjusted *P* < 0.05) and subjected to KEGG pathway enrichment analysis using the “clusterProfiler” R package. Furthermore, Gene Set Enrichment Analysis (GSEA) was employed to visualize and quantify the activity of representative signaling pathways.

### Cell–cell communication analysis

Intercellular communication networks were inferred using the “CellChat” R package. The CellChatDB.mouse database was utilized to predict ligand–receptor interactions among five major cell types: Macrophages, Monocytes, Alveolar Epithelial, Neutrophils, and Dendritic Cells. To ensure statistical robustness, interactions were filtered using a minimum expression cutoff of 0.1 and a minimum cell count of 10 per cell group.

### Recombinant NMI protein

Full-length recombinant mouse NMI (mNMI) and human NMI (hNMI) proteins were expressed in Escherichia coli strain BL21(DE3). The proteins were sequentially purified using a nickel-nitrilotriacetic acid (Ni-NTA) affinity column, followed by ion-exchange chromatography with a HiTrap Q HP column (GE Healthcare) and size-exclusion chromatography with a Superdex 200 column (GE Healthcare) using a fast protein liquid chromatography (FPLC) system. Protein purity was assessed by Coomassie Brilliant Blue staining and confirmed to be >95%. Endotoxin was strictly removed using a Polymyxin B (PMB; Thermo Fisher, Cat#88276) affinity column. The final endotoxin levels were verified to be < 1 EU/μg protein using a Limulus Amebocyte Lysate (LAL) assay kit (Andus, Cat# RTO65500). The purified proteins were formulated in phosphate-buffered saline (PBS), aliquoted, and stored at -80 °C. Furthermore, to definitively exclude the possibility of endotoxin-mediated artifacts during *in vitro* experiments, hNMI was subjected to overnight digestion with trypsin and used as an inactivated control.

### Cell culture and treatment

The human alveolar epithelial cell line A549 was obtained from the National Infrastructure of Cell Line Resource (China). Cells were maintained in Dulbecco’s Modified Eagle Medium (DMEM; Gibco) supplemented with 10% fetal bovine serum (FBS; Gibco), 100 IU/mL penicillin, and 100 µg/mL streptomycin (Gibco). All cultures were incubated at 37 °C in a humidified atmosphere containing 5% CO_2_.

To induce inflammatory activation, A549 cells were pretreated with the STAT3 inhibitor Stattic (100 nM; Selleck, S7024) for 2 hours, followed by stimulation with hNMI (5 µg/mL) for the indicated durations. Subsequently, whole cell lysates were harvested, and the protein expression levels of TF, STAT3, and phosphorylated STAT3 (p-STAT3) were determined by Western blotting.

### Reverse transcription quantitative PCR

Total RNA was extracted from A549 cells using the FreeZol Reagent kit (Vazyme, R711-01) and reverse-transcribed into cDNA using the StarScript III All-in-one RT Mix (Genstar, A230-10). Expression levels of the corresponding genes were then analyzed by quantitative real-time polymerase chain reaction using the following primer sequences. GAPDH was used as the reference gene.

*F3*: F 5’-TACAGACAGCCCGGTAGAGT-3’ R 5’-AGCTCCAACAGTGCTTCCTT-3’

*GAPDH*: F 5’-TTCCAGGAGCGAGATCCCT-3’ R 5’-CACCCATGACGAACATGGG-3’

### Poly(I:C)-induced pulmonary coagulopathy mouse model

Eight- to ten-week-old wild-type (WT) male C57BL/6 mice were purchased from SPF (Beijing) Biotechnology Co., Ltd. All mice were housed in a specific pathogen-free (SPF) facility at the Experimental Animal Center of Sun Yat-sen University. *Nmi* knockout (*Nmi*^-/-^) mice were generated using CRISPR-Cas9 technology as previously described (21).

To establish the poly(I:C)-induced viral pneumonia model, mice were randomly allocated into their respective experimental groups (n = 6 per group). WT and *Nmi*^-/-^ mice were intratracheally (i.t.) administered either poly(I:C) (10 mg/kg; Selleck, E0518) or an equal volume of sterile PBS. In the Poly(I:C) + Stattic group, mice received an intraperitoneal (i.p.) injection of Stattic (25 mg/kg) 1 h prior to the initial poly(I:C) challenge (27, 28). For the *in vivo* rescue experiment, poly(I:C) and mNMI (5 mg/kg) were mixed in sterile PBS and intratracheally injected simultaneously into *Nmi*^-/-^ mice.

At 24 h post-challenge, the mice were deeply anesthetized via the inhalation of 4-5% isoflurane in 100% O_2_ (1 L/min) and subsequently euthanized by cervical dislocation. Finally, bronchoalveolar lavage fluid (BALF) and lung tissues were collected for downstream analyses.

### Hematoxylin and eosin staining and histopathological analysis

H&E staining experiments were performed as previously reported (29). Mouse lung tissues were fixed in 4% paraformaldehyde for 24 hours, embedded in paraffin, and cut into 4-μm-thick sections. After deparaffinization and rehydration, the sections were stained with hematoxylin, differentiated with acid alcohol, and blued with Scott’s tap water substitute. Subsequently, the sections were counterstained with eosin, dehydrated through a graded ethanol series, and mounted with neutral gum. Images were captured using a Nikon microscope with a 20× objective.

To quantify lung injury, four random fields from the region of interest (ROI) were evaluated for each lung tissue sample by two independent investigators who were blinded to the experimental group allocations. The lung injury score was calculated based on the following five parameters: 1) neutrophils in the alveolar space, 2) neutrophils in the interstitial space, 3) hyaline membranes, 4) proteinaceous debris filling the airspaces, and 5) alveolar septal thickening. The weighted sum of these five independent variables was normalized to the number of fields evaluated, yielding a continuous injury score between 0 and 1 (inclusive), as recommended by the American Thoracic Society guidelines (30).

### Immunofluorescent staining and imaging

The paraffin-embedded lung sections were rinsed with PBS, followed by permeabilization and blocking with 5% goat serum for 30 minutes at room temperature. The sections were then incubated overnight at 4 °C with primary antibodies diluted in PBS. After washing three times with PBS (10 min each), the sections were incubated with fluorescently labeled secondary antibodies and DAPI for 1 h at room temperature. Following three additional washes, the sections were mounted using ProLong mounting medium. Images were acquired using a fluorescence microscope (Nikon) equipped with 10×, 20×, and 40× objectives. The primary antibodies used in this study included anti-F4/80 (1:500, Servicebio, #GB113373), anti-Ly6G (1:200, Servicebio, #GB11229), and anti-Fibrinogen (1:100, Servicebio, #GB112088).

For quantitative image analysis, at least three randomly selected high-power fields per section were evaluated. ImageJ software (National Institutes of Health, Bethesda, MD, USA) was used to quantify the expression levels of fibrinogen and inflammatory markers by calculating the percentage of the positive staining area relative to the total tissue area within each field. To eliminate observer bias, all image acquisitions and quantitative analyses were performed by independent investigators in a blinded manner.

### Western blot

Lung tissue samples or A549 cells were homogenized in RIPA lysis buffer (Beyotime, P0013C) supplemented with a protease and phosphatase inhibitor cocktail. Lysates were incubated on ice for 30 min and subsequently centrifuged at 12,000 × g for 15 min at 4°C to collect the supernatant. Protein concentration was quantified using a BCA Protein Assay Kit (Beyotime, P0012S). Equal amounts of protein (30–50 μg) were resolved by 10% SDS-PAGE and electrophoretically transferred onto polyvinylidene fluoride (PVDF) membranes. Membranes were blocked with 5% non-fat dry milk in Tris-buffered saline containing 0.1% Tween-20 (TBST) for 1 h at room temperature. The membranes were then incubated overnight at 4 °C with the following primary antibodies: anti-NMI (1:1000; Servicebio, GB114586-100), anti-TF (1:1000; Abcam, #RM1198), anti-STAT3 (1:1000; CST, #12640), anti-p-STAT3 (Tyr705; 1:1000; CST, #9145), and anti-GAPDH (1:10,000; Huabio, #ET1601-4). Following three washes with TBST, the membranes were incubated with HRP-conjugated secondary antibodies (1:5000) for 1 h at room temperature. Protein bands were visualized using enhanced chemiluminescence (ECL) reagents (Vazyme, E423) and imaged using a chemiluminescence detection system (Bio-Rad, Hercules, CA, USA). Band intensities were quantified using ImageJ software (NIH, Bethesda, MD, USA), with protein expression levels normalized to GAPDH. All experiments were performed in triplicate.

### ELISA

BALF samples were collected and centrifuged at 1,500 × g for 10 min at 4 °C to remove debris. Concentrations of IL-6 (DaYou, Cat#DKW12-2060-048), TNF-α (DaYou, Cat#1217202), TAT (Cusabio, #CSB-E08433m), PAI-1 (Enova, #E-HS30773Mo) and TF (Enova, #EN-32335Mo) were quantified using commercial ELISA kits according to the manufacturers’ protocols. Briefly, 100 μL of standards or samples were added to 96-well plates pre-coated with specific capture antibodies and incubated at 37 °C for 1–2 h. After washing, wells were incubated with biotinylated detection antibodies, followed by HRP-conjugated streptavidin. Color development was performed using TMB substrate, and the reaction was stopped with stop solution. Absorbance was measured at 450 nm using a microplate reader (BioTek). All measurements were performed in triplicate, and cytokine concentrations were calculated based on standard curves generated from known concentrations of recombinant proteins.

### Statistical analysis

Bioinformatics analyses were conducted in R (v4.4.1). Experimental data were analyzed using an unpaired two-tailed Student’s *t*-test, a one-way ANOVA followed by Tukey’s multiple comparisons test, or the Wilcoxon rank-sum test in Prism 8 (GraphPad). Data are expressed as mean ± SEM. *p* < 0.05 was considered statistically significant.

## Results

### NMI is robustly upregulated in viral pneumonia and correlates with coagulation signatures

**Figure 1:**
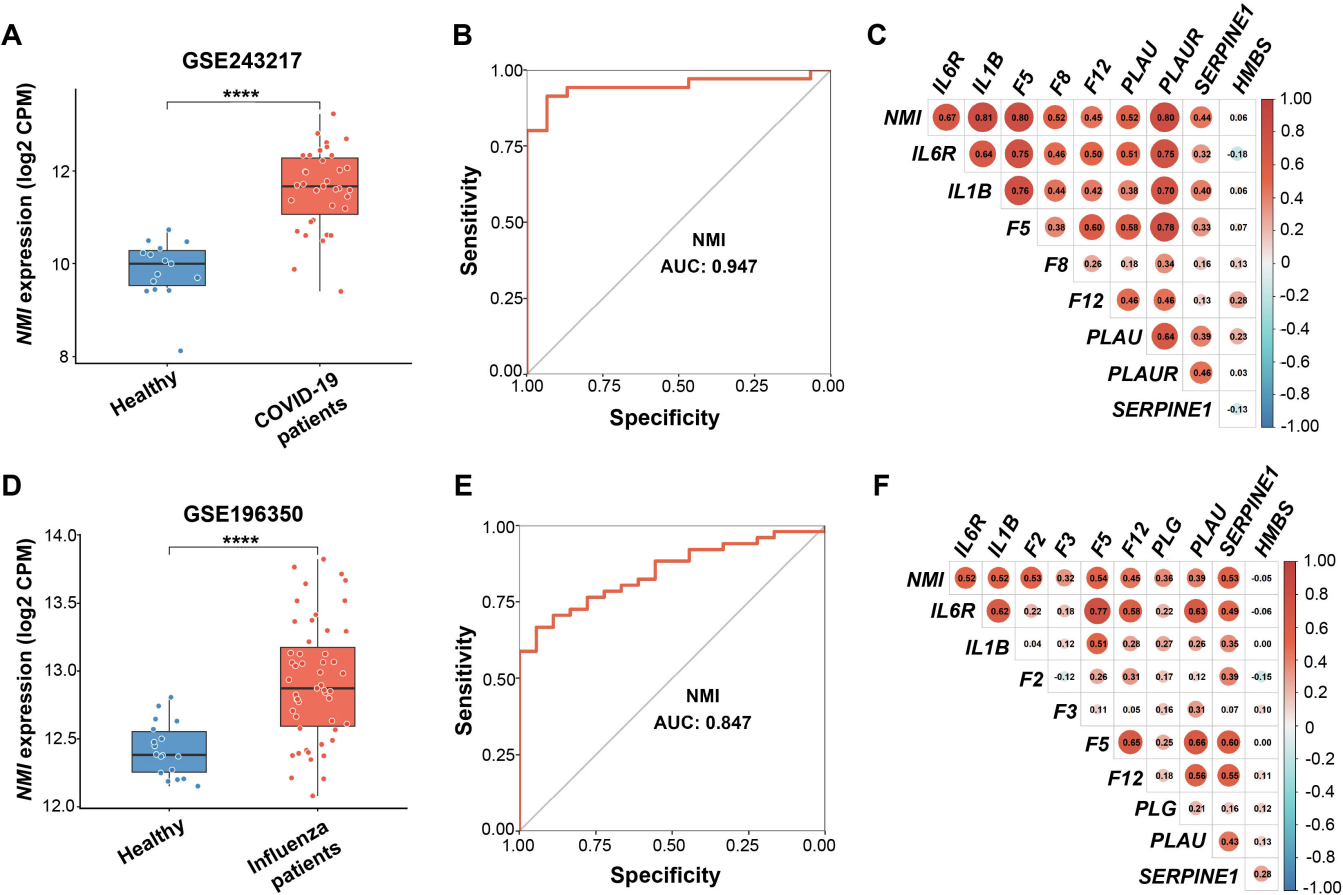
*NMI* is significantly upregulated in viral pneumonia patients and demonstrates discriminative potential within analyzed cohorts. **(A)** Expression levels of *NMI* in peripheral blood samples from COVID-19 patients compared to healthy controls (GSE243217). **(B)** ROC analysis evaluating the discriminative performance of *NMI* in distinguishing COVID-19 patients from healthy controls, with an AUC of 0.947. **(C)** Correlation analysis between *NMI* expression and key pro-inflammatory cytokines, coagulation factors, and the housekeeping gene *HMBS* in peripheral blood from COVID-19 patients. **(D)** *NMI* expression in peripheral blood from influenza patients and healthy donors (GSE196350). **(E)** ROC curve analysis evaluating the discriminative performance of *NMI* in distinguishing influenza patients from healthy controls, with an AUC of 0.847. **(F)** Correlation analysis between *NMI* expression and key pro-inflammatory cytokines, coagulation factors, and the housekeeping gene *HMBS* in peripheral blood from influenza patients. Statistical significance was determined using an unpaired two-tailed Student’s *t*-test. ****p < 0.0001. Correlations were assessed using Pearson correlation coefficients.

To investigate the clinical relevance of NMI in viral pneumonia, we analyzed transcriptomic datasets from both human patients and murine models. In cohorts of COVID-19 and influenza-infected patients, *NMI* mRNA levels were significantly upregulated in peripheral blood (**Figures 1A, D**), suggesting a robust systemic response to viral infection. Corroborating these clinical findings, we observed a parallel elevation of *Nmi* expression in the lung tissues of mice infected with H1N1 influenza virus or respiratory syncytial virus (RSV) (**Supplementary Figures 1A, C**).

To evaluate the potential diagnostic value of NMI, we performed receiver operating characteristic (ROC) curve analyses. Circulating NMI levels demonstrated promising discriminative ability, distinguishing COVID-19 patients from healthy controls with an area under the curve (AUC) of 0.947 (**Figure 1B**) and influenza patients with an AUC of 0.847 (**Figure 1E**).

We next sought to determine whether NMI expression is linked to the pathological coagulopathy characteristic of severe infections. Co-expression analyses revealed that NMI was positively associated with key mediators of the coagulation cascade across multiple viral etiologies. Specifically, *NMI* showed significant positive correlations with critical coagulation and fibrinolysis-related genes, including *F2*, *F3*, *F5*, *F8*, *F12*, *PLAU*, *PLAUR*, *PLG*, and *SERPINE1*, alongside markers of disease severity such as *IL6R* and *IL1B* (**Figures 1C, F**). To validate the specificity of this pro-coagulant transcriptional signature, the standard housekeeping gene *HMBS* was evaluated as a parallel control and exhibited no such significant correlation with *NMI*. Importantly, this transcriptional signature was conserved in murine models, where *Nmi* exhibited strong positive correlations with coagulation factors and inflammatory markers in both influenza (**Supplementary Figure 1B**) and RSV (**Supplementary Figure 1D**) infections. Similarly, the standard murine housekeeping gene *Rps16* was evaluated as a parallel control and exhibited no significant correlation with *Nmi*. Collectively, these cross-species data consistently link the NMI axis to the activation of the coagulation cascade, underscoring its potential role as a potential contributor to viral pneumonia-associated coagulopathy.

### NMI correlates with alveolar epithelial hyperactivation in viral pneumonia

Given that alveolar barrier injury is central to the pathogenesis of viral coagulopathy, we utilized single-sample gene set enrichment analysis (ssGSEA) to profile the cellular landscape of 40 distinct cell types in influenza-infected mice (GSE271812). Our analysis revealed a profound remodeling of the lung microenvironment linked to tissue damage (**Supplementary Figure 2A**). Specifically, we observed a significant enrichment of the epithelial cell signature, indicative of epithelial hyperactivation or hyperplasia in response to viral insults. Concurrently, we noted the expansion of infiltrating myeloid subsets (monocytes and macrophages), depicting a lung microenvironment defined by severe stress and inflammatory infiltration (**Supplementary Figure 2B**).

**Figure 2:**
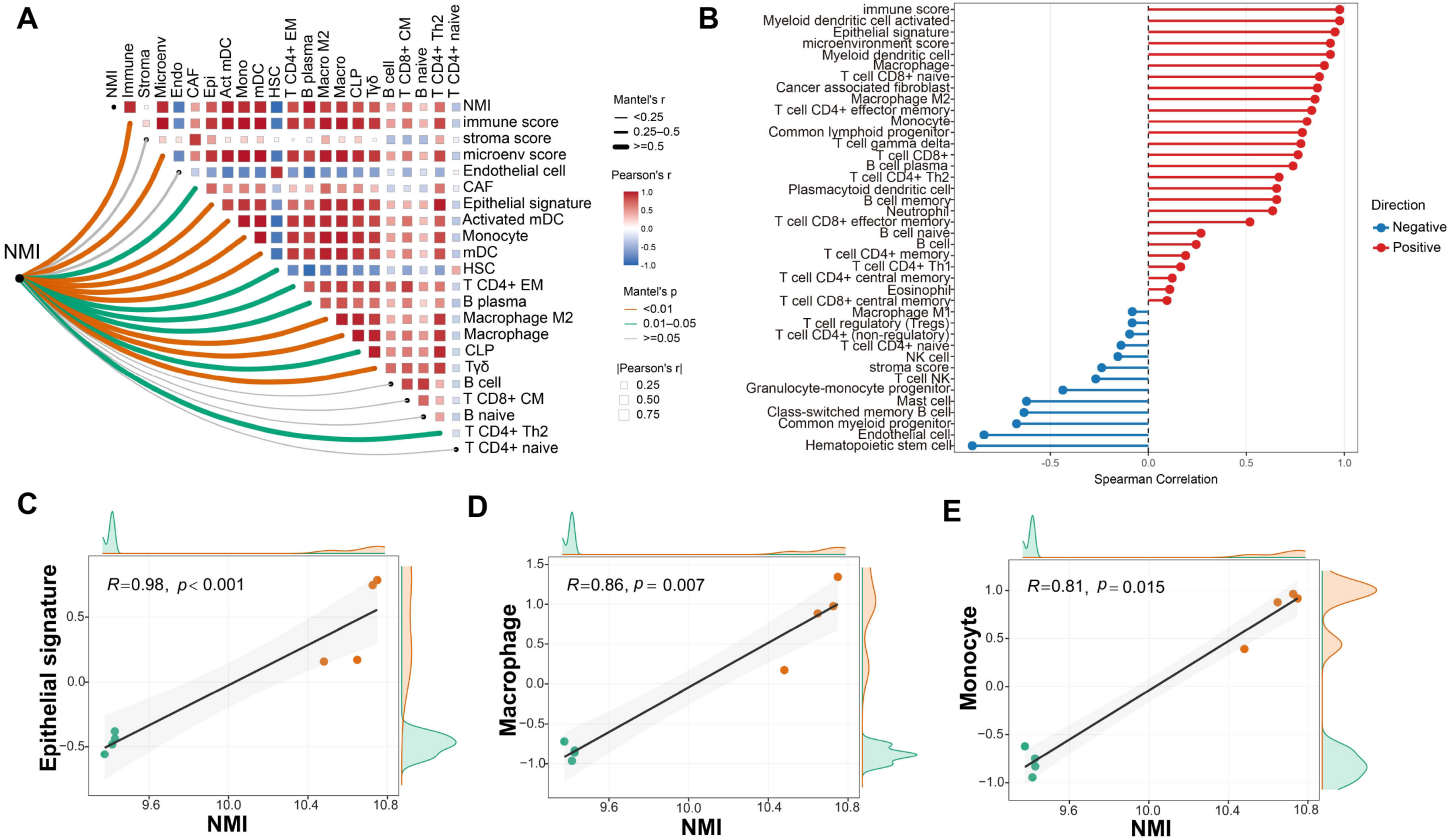
*Nmi-*associated epithelial transcriptional hyperactivation and immune recruitment in influenza pneumonia. **(A)** A Mantel test and correlation analysis illustrating the relationship between *Nmi* expression and the lung cellular landscape. The heatmap displays pairwise correlations between cell signatures, while connecting lines indicate the strength (Mantel’s *r*) and significance (Mantel’s *p*) of associations with *Nmi*. **(B)** Lollipop chart ranking cell populations based on their Spearman correlation coefficients with *Nmi*, highlighting the specific enrichment of pro-inflammatory subsets (red lollipops) versus depleted populations (blue lollipops). **(C–E)** Scatter plots validating the robust associations between *Nmi* expression and specific cell signatures (n=8). *Nmi* levels were significantly positively correlated with **(C)** the epithelial signature, **(D)** macrophages, and **(E)** monocytes. Green dots represent the control group (PBS), and orange dots represent the viral pneumonia group (IAV). Pearson correlation coefficients (R) and *p*-values are indicated in each panel.

We next investigated the association between *Nmi* expression and this cellular remodeling. A Mantel test and Spearman analyses revealed that *Nmi* was intimately linked to the degree of lung pathology (**Figures 2A, B**). Most notably, *Nmi* displayed a striking positive correlation with the epithelial signature (*R* = 0.98) (**Figure 2C**), strongly suggesting that *Nmi* expression is tightly linked to the activated epithelial state. Strong correlations were also observed with infiltrating myeloid lineages (macrophages, *R* = 0.86; monocytes, *R* = 0.81) (**Figures 2D, E**), likely reflecting the recruitment of these cells to sites of severe epithelial damage. Collectively, these findings suggest that *Nmi* upregulation is intrinsically coupled with alveolar epithelial hyperactivation, creating a microenvironment prone to pathological coagulation.

### NMI expression is coupled with JAK-STAT signaling and coagulation cascades

**Figure 3:**
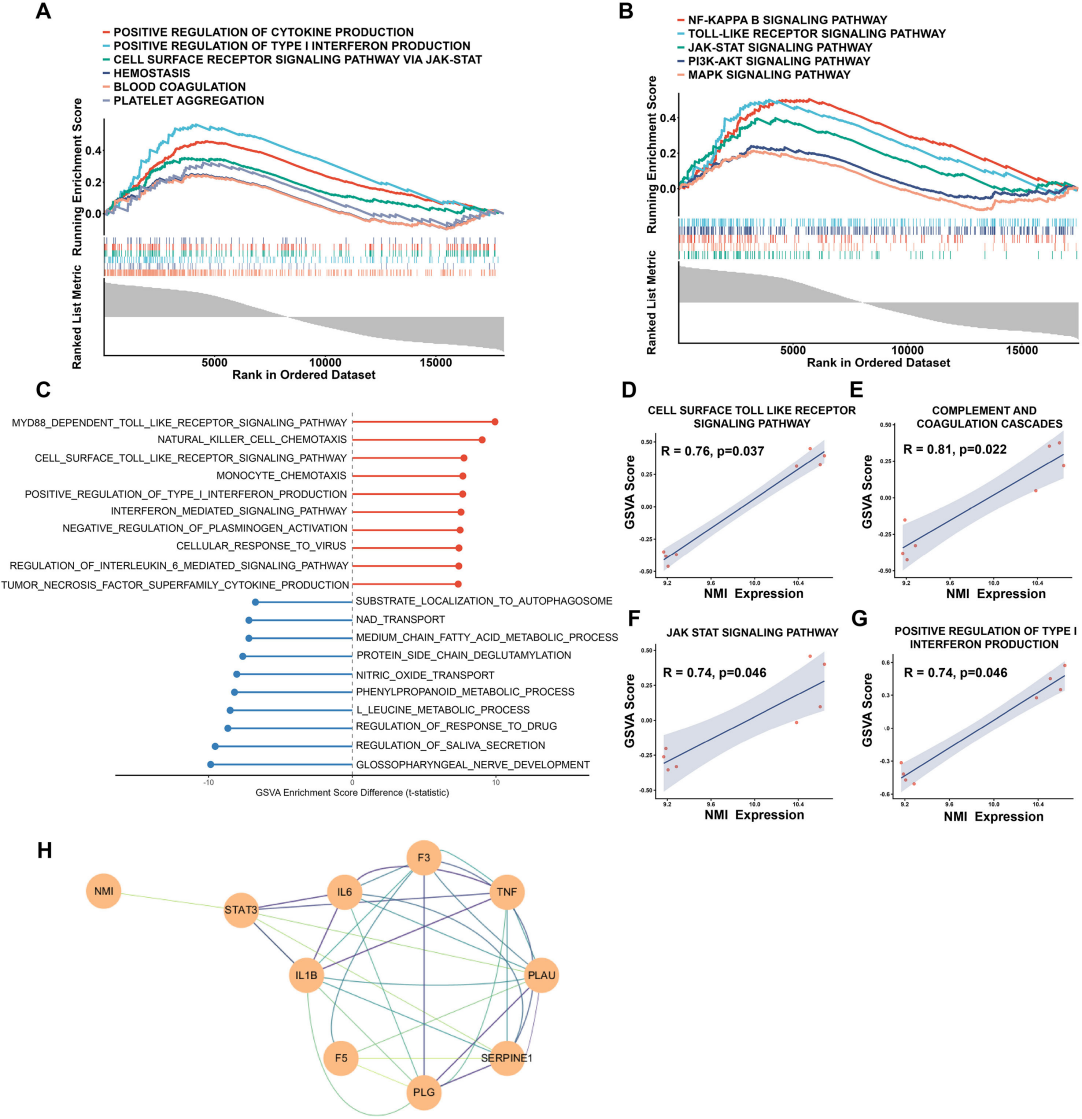
Functional enrichment and pathway analysis reveal *Nmi* as a key regulator of inflammation and coagulation. **(A)** GSEA of GO terms positively correlated with *Nmi* (GSE271812). The top biological processes are ranked by significance. **(B)** GSEA of KEGG pathways identifying signaling cascades associated with *Nmi* expression. **(C)** Lollipop chart illustrating the top upregulated (positive enrichment score) and downregulated (negative enrichment score) pathways in the viral pneumonia dataset. **(D–G)** Scatter plots illustrating the robust positive correlations between *Nmi* expression and key immune and coagulation-related signatures. **(H)** STRING interaction network revealing the molecular crosstalk between NMI and the STAT3-driven coagulation module.

To elucidate the molecular mechanisms underlying the strong association between *Nmi* upregulation and epithelial hyperactivation, we identified genes positively correlated with *Nmi* expression in lung tissues of influenza-infected mice (GSE271812) and performed Gene Set Enrichment Analysis (GSEA) using Gene Ontology (GO) terms. This analysis revealed a significant enrichment of biological processes critical for thrombogenesis, including hemostasis, platelet aggregation, and blood coagulation. Notably, terms related to the JAK-STAT signaling pathway were also robustly enriched among these *Nmi*-co-expressed genes (**Figure 3A**).

Complementary KEGG pathway analysis via GSEA further corroborated these findings, highlighting strong associations with key signaling cascades including the Toll-like receptor (TLR), NF-κB, PI3K-Akt, and MAPK pathways (**Figure 3B**). While these pathways are canonical mediators of the antiviral response, their robust co-enrichment highlights a signaling landscape where NMI is intimately coupled with stress-response kinases capable of modulating downstream coagulation targets.

To further contextualize these findings, we applied GSVA to quantify pathway activity variations between control and influenza-infected mice. Among the top 200 differentially active pathways, with 185 upregulated and 15 downregulated, the key pathways intimately linked to the disease phenotype, particularly those governing coagulation and immune responses, are displayed in **Figure 3C**. Correlation analysis subsequently identified four pathways most strongly associated with *Nmi* expression: the “Cell surface Toll-like receptor signaling pathway”, “Complement and coagulation cascades”, “JAK-STAT signaling pathway”, and “Positive regulation of type I interferon production” (**Figures 3D–G**).

Notably, these diverse analyses converged on a common regulatory hub: the JAK-STAT signaling pathway. Given that STAT3 is a pivotal transcription factor within this family, widely recognized as a direct transcriptional activator of tissue factor (*F3*) (31–33), we hypothesized that STAT3 serves as the critical effector bridging NMI signaling to the coagulation cascade. To test this, we constructed a Protein-protein interaction (PPI) network using the STRING database and visualized it via Cytoscape. The analysis highlighted a tight functional cluster linking NMI, STAT3, and key coagulation factors (PLAU, SERPINE1, F3) (**Figure 3H**).

Collectively, these findings suggest that NMI is functionally positioned to contribute to pathological coagulation, potentially promoting the upregulation of thrombotic factors via the JAK-STAT3 axis.

### Single-cell analysis identifies activated alveolar epithelial cells as a critical source of secreted NMI

**Figure 4:**
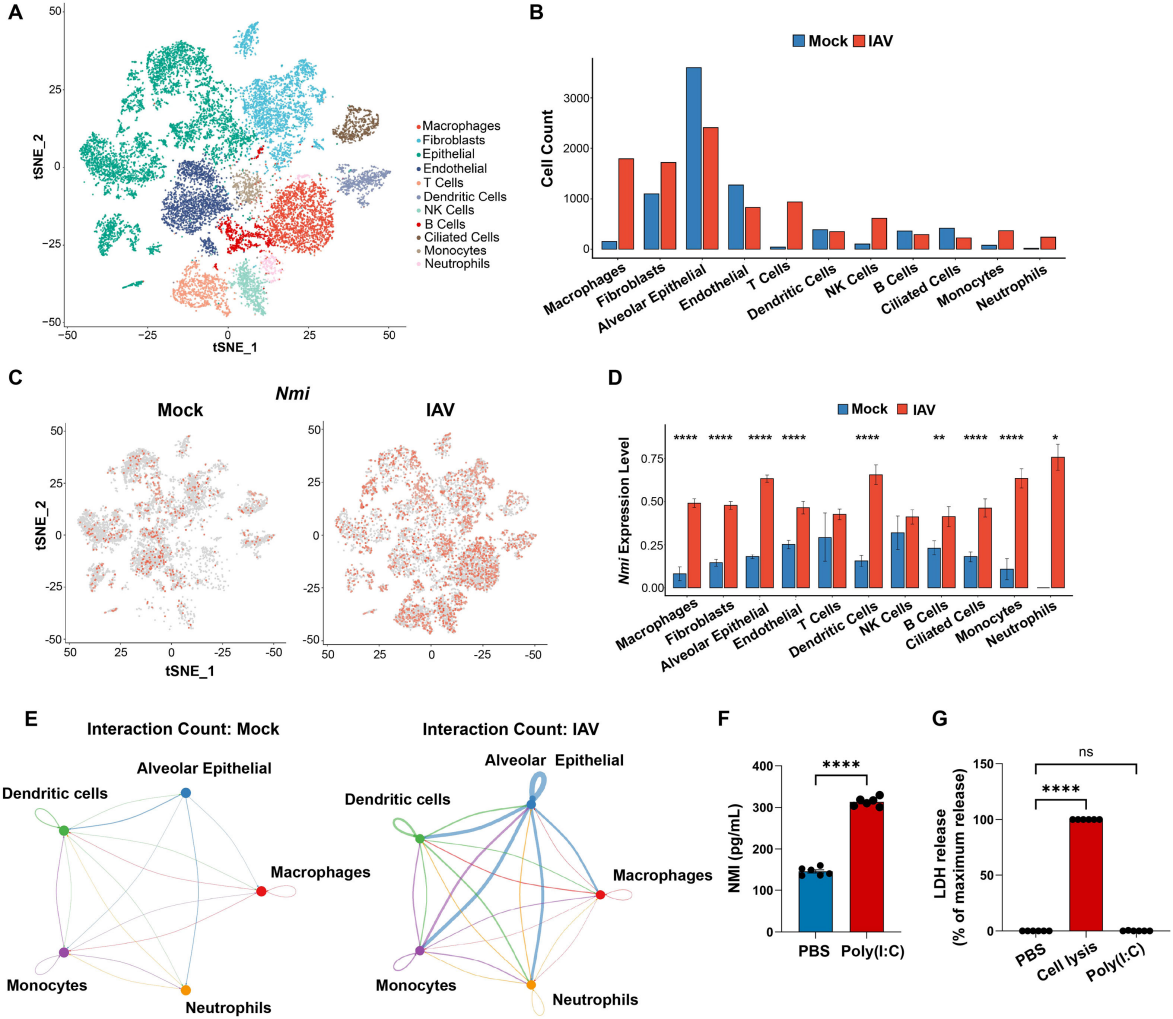
Single-cell transcriptomic atlas reveals *Nmi* upregulation and cellular crosstalk in influenza-infected lungs. **(A)** t-SNE visualization of 17,276 single cells from mouse lung tissues (Dataset: GSE292515), resolved into 23 clusters and color-coded by the 11 major annotated cell types. **(B)** Bar plot comparing the absolute cell counts of each cell population between the Mock and Influenza A virus (IAV) infected groups. **(C)** Feature plot projecting *Nmi* expression onto the t-SNE map. Red color intensity indicates expression levels. **(D)** Quantitative expression of *Nmi* across all annotated cell types. Note the significant upregulation of *Nmi* in alveolar epithelial cells and the myeloid compartment (including neutrophils, dendritic cells, monocytes, and macrophages). **(E)** Cell-cell communication network analysis showing the interaction counts among major cell populations in Mock and IAV groups. Line thickness represents the number of inferred ligand-receptor interactions, highlighting the signaling connectivity from myeloid lineages to epithelial cells. **(F)** Validation of NMI secretion *in vitro*. A549 cells were stimulated with poly(I:C) to mimic viral infection, and NMI levels in the culture supernatant were measured by ELISA. **(G)** A549 cells were treated with PBS or poly(I:C). Cytotoxicity was evaluated by measuring the release of LDH into the cell culture supernatant. Cells treated with the lysis buffer provided in the assay kit served as the positive control for maximum LDH release (defined as 100%). Data are presented as mean ± SEM. Statistical significance was determined using an unpaired two-tailed Student’s *t*-test or one-way ANOVA. ns, not significant, *p < 0.05, **p < 0.01, ****p < 0.0001.

To validate our transcriptomic findings in a granular cellular context, we analyzed single-cell RNA-seq (scRNA-seq) data from control and influenza-infected mouse lungs (GSE292515), comprising 17,276 high-quality cells. Unsupervised clustering and dimensionality reduction identified 23 transcriptionally distinct clusters, which were annotated using canonical marker genes (**Supplementary Figures 3A, B**) and consolidated into 11 major lineages: fibroblasts, macrophages, monocytes, alveolar epithelial cells (including alveolar type I/II), endothelial cells, T cells, NK cells, B cells, dendritic cells, ciliated cells, and neutrophils (**Figure 4A**). Comparative analysis of cell composition revealed a dramatic remodeling of the lung ecosystem upon infection: a marked expansion of inflammatory infiltrates was observed alongside a severe depletion of structural barrier populations, specifically alveolar epithelial and endothelial cells (**Figure 4B**; **Supplementary Figure 3C**). This cellular shift highlights the profound loss of alveolar-capillary barrier integrity, a hallmark microenvironment primed for pathological coagulation.

To determine the cellular contributors to NMI-driven coagulopathy, we first mapped the expression distribution of *Nmi*. While *Nmi* exhibited a broad distribution across immune and structural subsets, it was most robustly induced in infiltrating myeloid lineages (macrophages, dendritic cells, monocytes, and neutrophils), and alveolar epithelial cells (**Figures 4C, D**). Given that these cell types are recognized as key effectors of coagulation and tissue injury, we performed cell-cell communication analysis to dissect their interactions. In the viral infection group, we observed a marked enhancement in signaling flux from myeloid cells to alveolar epithelial cells. Crucially, the autocrine signaling loop within alveolar epithelial cells was also significantly amplified (**Figure 4E**). This finding, combined with our previous knowledge that activated macrophages secrete NMI (21, 22), prompted us to investigate whether alveolar epithelial cells also possess the capacity to release NMI into the extracellular milieu. To experimentally validate this, we stimulated A549 cells (a model of alveolar type II epithelial cells) with the viral mimetic poly(I:C) (50 μg/mL) (34, 35). Consistent with the transcriptional upregulation observed in our single-cell data, we detected a robust release of NMI protein into the culture supernatant (**Figure 4F**). To exclude the possibility that this extracellular accumulation was merely a passive consequence of cell lysis, we evaluated cellular viability using a lactate dehydrogenase (LDH) release assay. Importantly, poly(I:C) stimulation did not induce significant cytotoxicity in A549 cells (**Figure 4G**). These results confirm that, rather than leaking passively from dead cells, activated epithelial cells actively secrete NMI upon viral sensing, identifying them as a critical source of extracellular NMI in the lung microenvironment.

### Extracellular NMI triggers STAT3-dependent tissue factor expression in the alveolar epithelium

**Figure 5:**
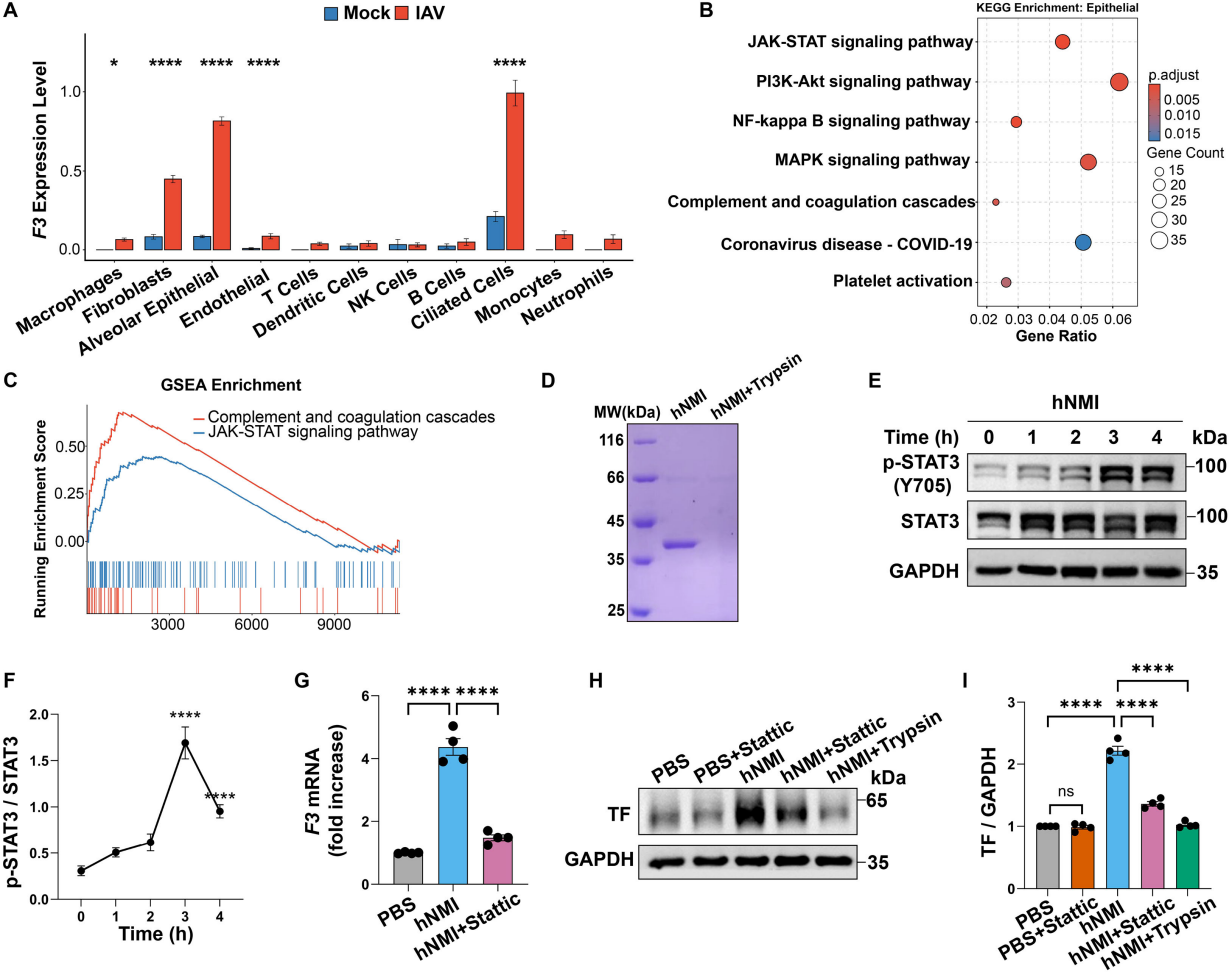
Extracellular NMI promotes tissue factor expression in the alveolar epithelium via the STAT3 signaling axis. **(A)** Quantitative expression profile of *F3* across all annotated lung cell clusters, highlighting its significant enrichment in the epithelial compartment (alveolar epithelial cells and ciliated cells). **(B)** KEGG pathway enrichment analysis of alveolar epithelial cells, stratified by high versus low *Nmi* expression levels. **(C)** GSEA plots demonstrating the significant enrichment of the JAK-STAT signaling pathway and complement and coagulation cascades in *Nmi*-high alveolar epithelial cells. **(D)** SDS-PAGE analysis and Coomassie blue staining of hNMI protein used for A549 cell activation. hNMI protein was inactivated by incubation with trypsin at a final concentration of 250 μg/mL overnight at 37 °C to serve as a control. **(E)** Western blot analysis of STAT3 phosphorylation (p-STAT3) and total STAT3 in A549 cells stimulated with hNMI at the indicated time points (0–4 h). **(F)** Densitometric quantification of the p-STAT3/total STAT3 ratio over time. **(G)** RT-qPCR analysis of *F3* mRNA levels in A549 cells treated with hNMI in the presence or absence of the STAT3 inhibitor Stattic (100 nM). **(H–I)** Western blots and quantitative analysis of TF protein expression. Cells were treated with hNMI, hNMI + Stattic, or trypsin-inactivated hNMI. Note that Stattic abolished NMI-induced TF upregulation, while inactivated NMI failed to induce TF expression. Data are presented as mean ± SEM. Statistical significance was determined using an unpaired two-tailed Student’s *t*-test or one-way ANOVA. ns, not significant, *p < 0.05, ****p < 0.0001.

To pinpoint the cellular origin of the coagulation cascade, we mapped the expression landscape of *F3*. In contrast to the broad distribution of *Nmi*, *F3* expression was spatially restricted and predominantly upregulated in the epithelial compartment, particularly in alveolar and ciliated cells (**Figure 5A**). Given our finding that activated epithelial cells actively secrete NMI, this co-localization of *Nmi* and *F3* suggests a potential autocrine amplification loop: secreted NMI may act directly on the epithelium to induce TF expression and initiate coagulation.

To dissect the intracellular signaling landscape driven by NMI specifically within the epithelial compartment, we stratified single-cell epithelial populations into *Nmi*-high and *Nmi*-low subsets. Differential pathway activation analysis revealed distinct molecular signatures. KEGG enrichment analysis highlighted the preferential activation of the JAK-STAT signaling pathway in *Nmi*-high cells, alongside PI3K-Akt, NF-κB, MAPK, and complement and coagulation cascades (**Figure 5B**). Consistently, GSEA identified the JAK-STAT signaling signature and coagulation cascades as the top-ranked enriched pathways (**Figure 5C**). To spatially visualize this relationship, we projected the JAK-STAT signaling score onto the t-SNE embedding. Notably, we observed a striking overlap: alveolar epithelial clusters expressing high levels of *F3* exhibited a concurrent elevation in JAK-STAT signaling activity (**Supplementary Figures 4A, B**), further supporting the regulatory link between this pathway and TF expression. Collectively, these transcriptomic data strongly suggest that NMI expression is intrinsically coupled with JAK-STAT pathway activation within the epithelial compartment, positioning the NMI-JAK-STAT-TF axis as a critical upstream modulator of the coagulation cascade.

To substantiate the causal link suggested by our transcriptomic analyses, we utilized A549 cells as an *in vitro* model. Cells were treated with purified recombinant human NMI (hNMI) expressed in E. coli to recapitulate the extracellular DAMP microenvironment. To rule out potential confounding effects from endotoxin contamination, hNMI was inactivated via overnight trypsin digestion and utilized as a negative control (**Figure 5D**). Given that STAT3 signaling is a known modulator of acute lung injury and a transcriptional activator of TF (36), we hypothesized that NMI operates through this axis. Consistent with our hypothesis, extracellular hNMI stimulation triggered rapid and robust STAT3 phosphorylation (p-STAT3), peaking at 3 hours post-stimulation (**Figures 5E, F**). Subsequently, hNMI treatment significantly upregulated TF at both the mRNA and protein levels by 24 hours (**Figures 5G–I**). Crucially, this induction was effectively abrogated by pharmacological inhibition of STAT3 using Stattic. Importantly, a CCK-8 assay confirmed that Stattic treatment (100 nM) did not affect A549 cell viability across a 48-hour period (**Supplementary Figure 4C**), verifying that the observed TF suppression is strictly driven by STAT3 signaling blockade rather than drug-induced cytotoxicity. To confirm signaling specificity, we demonstrated that trypsin-digested hNMI failed to elicit these responses. Collectively, these results establish the NMI-STAT3-TF axis as an important contributor to epithelial pro-coagulant activation, providing a mechanistic basis for targeting this pathway.

### Genetic ablation or pharmacological inhibition of the NMI-STAT3 axis mitigates poly(I:C)-induced lung injury and coagulopathy

To functionally validate the pathogenic role of NMI *in vivo*, we established a murine model of viral pneumonia using intratracheal administration of poly(I:C). As a synthetic analog of viral double-stranded RNA, poly(I:C) serves as a potent agonist of Toll-like receptor 3 (TLR3). This model is widely recognized for its ability to recapitulate the severe inflammatory response and pathological features characteristic of acute viral infections (37–39).

Prior to disease modeling, we first validated our genetic and experimental tools. WT and *Nmi*^-/-^ mice were confirmed via PCR genotyping of tail tissue DNA (**Supplementary Figure 5A**). Importantly, baseline histological evaluation using H&E staining revealed that *Nmi* deletion alone neither altered normal lung architecture nor induced spontaneous inflammatory infiltration (**Supplementary Figure 5B**). For the *in vivo* rescue experiments, we expressed and purified recombinant murine NMI (mNMI) using an E. coli expression system (**Supplementary Figure 5C**). To strictly rule out potential confounding inflammatory responses caused by bacterial contaminants, the purified protein underwent rigorous endotoxin removal. Following these validations, we evaluated the pathogenic role of NMI *in vivo* by comparing five experimental cohorts: (1) PBS-treated WT controls, (2) Poly(I:C)-treated WT mice (WT + Poly(I:C)), (3) Poly(I:C)-treated *Nmi*^-/-^ mice (*Nmi*^-/-^ + Poly(I:C)), (4) *Nmi*^-/-^ + Poly(I:C) mice rescued with mNMI (*Nmi*^-/-^ + Poly(I:C) + mNMI), and (5) WT + Poly(I:C) mice treated with Stattic (WT + Poly(I:C) + Stattic).

**Figure 6:**
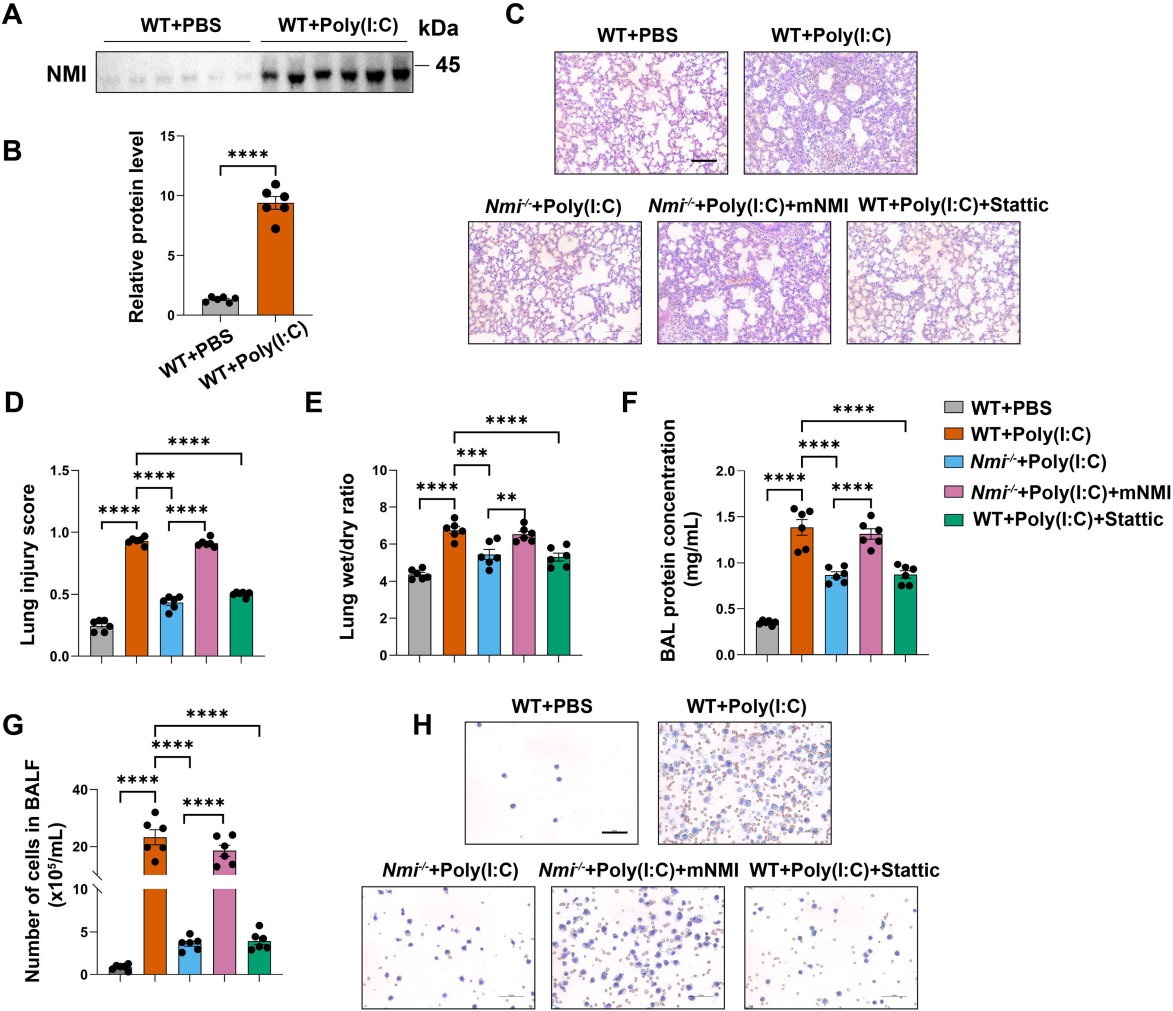
Targeting NMI attenuates lung inflammation and immune cell infiltration in a poly(I:C)-induced viral pneumonia model. **(A, B)** NMI protein levels in the BALF of WT mice challenged with poly(I:C) compared to PBS controls. **(C)** Representative H&E staining of lung sections. Groups include: WT+PBS, WT + Poly(I:C), *Nmi*^-/-^ + Poly(I:C), *Nmi*^-/-^ + Poly(I:C) + mNMI, and WT + Poly(I:C) + Stattic. Scale bars, 50 μm. **(D)** Histological lung injury scores, **(E)** lung wet-to-dry weight ratios, **(F)** total protein concentration in BALF, and **(G)** total cell counts in BALF were analyzed across the indicated groups. **(H)** Representative Wright-Giemsa staining of BALF smears, highlighting inflammatory cell morphology. Scale bar, 25 μm. Each dot represents one mouse. Data are presented as mean ± SEM. Statistical significance was determined using an unpaired two-tailed Student’s *t*-test or one-way ANOVA. **p < 0.01, ***p < 0.001, ****p < 0.0001.

First, we confirmed that poly(I:C) challenge triggered significant secretion of NMI into the alveolar space, validating its role as an extracellular DAMP (**Figures 6A, B**). This release was accompanied by severe acute lung injury. H&E and lung wet/dry weight ratio analysis revealed that WT+Poly(I:C) mice developed pronounced alveolar septal thickening, hemorrhage, and pulmonary edema (**Figures 6C–E**). Consistent with barrier dysfunction, total protein concentration in BALF was markedly elevated (**Figure 6F**). Strikingly, both *Nmi* deletion and Stattic treatment significantly attenuated these pathological features. Importantly, the administration of mNMI to *Nmi*^-/-^ mice reversed this protection, restoring severe lung injury and edema, thereby confirming the causality of extracellular NMI.

We next assessed the cellular inflammatory response. Poly(I:C) challenge induced a massive influx of innate immune cells into the alveolar space, as evidenced by total cell counts and Wright-Giemsa staining (**Figures 6G, H**). This recruitment of innate immune cells, particularly macrophages and neutrophils, was corroborated by immunofluorescence staining (**Supplementary Figures 5D–F**). Importantly, consistent with the lung injury phenotypes, both *Nmi* deletion and pharmacological inhibition of STAT3 with Stattic significantly attenuated this poly(I:C)-induced immune cell infiltration. Conversely, the administration of mNMI to *Nmi*^-/-^ mice reversed this protective effect, dramatically restoring the massive recruitment of inflammatory cells to the lungs. Collectively, these findings demonstrate that targeting the NMI-STAT3 axis effectively attenuates lung inflammation and immune cell infiltration in a poly(I:C)-induced viral pneumonia model.

**Figure 7:**
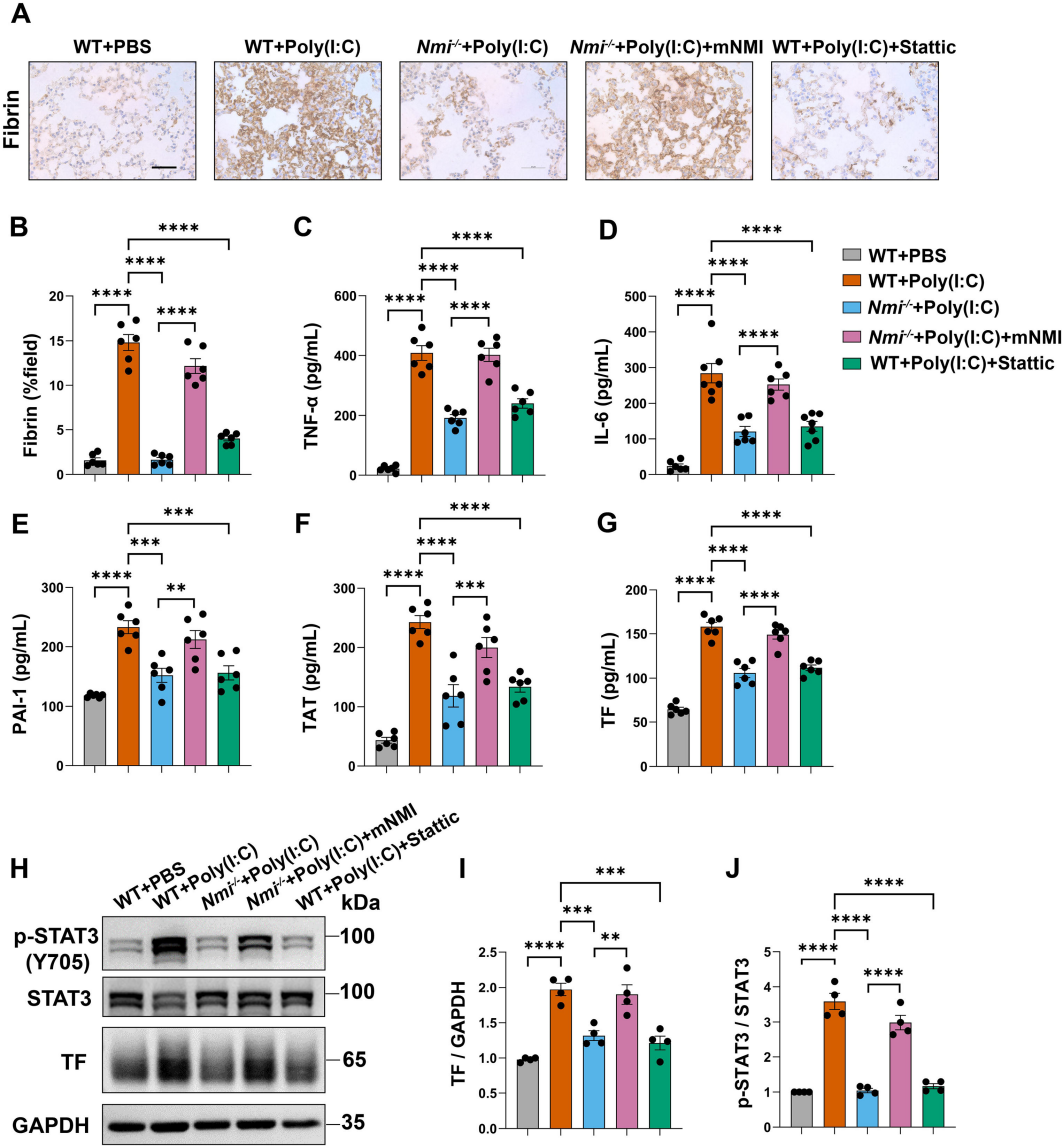
Targeting NMI alleviates the coagulation cascade in the poly(I:C)-induced viral pneumonia model. **(A, B)** Representative images and quantitative analysis of immunohistochemical staining for fibrin deposition in lung sections from the indicated groups. Scale bar, 25 μm. **(C–G)** Levels of pro-inflammatory cytokines **(C)** TNF-α and **(D)** IL-6, and coagulation mediators **(E)** PAI-1, **(F)** TAT, and **(G)** TF in BALF. **(H–J)** Western blot analysis and densitometric quantification of p-STAT3, total STAT3, and TF protein expression in lung tissues from the indicated groups. Each dot represents an individual mouse. Data are presented as mean ± SEM. Statistical significance was determined using one-way ANOVA. **p < 0.01, ***p < 0.001, ****p < 0.0001.

We next evaluated the downstream consequences on coagulation and systemic inflammation. Immunohistochemical staining demonstrated extensive fibrin deposition in the lungs of WT+Poly(I:C) and mNMI-rescue mice, which was largely absent in the *Nmi^-^*^/-^ and Stattic-treated groups (**Figures 7A, B**). Quantitative analysis of the BALF further confirmed that *Nmi* deficiency or STAT3 inhibition significantly suppressed the release of pro-inflammatory cytokines (TNF-α and IL-6) and attenuated the hypercoagulable state, as evidenced by reduced levels of thrombin-antithrombin (TAT) complexes, plasminogen activator inhibitor-1 (PAI-1), and TF (**Figures 7C–G**).

Finally, to elucidate the molecular signaling underlying this thrombo-inflammatory cascade, we analyzed lung homogenates. Western blot analysis revealed that the poly(I:C) challenge induced robust STAT3 phosphorylation alongside marked TF upregulation. Crucially, disruption of this pathway, either via genetic *Nmi* deletion or pharmacological STAT3 inhibition, abrogated both STAT3 activation and TF expression. Conversely, the administration of recombinant mNMI reversed this blockade and restored the signaling axis (**Figures 7H–J**). Collectively, these *in vivo* findings demonstrate that extracellular NMI mediates poly(I:C)-induced pulmonary coagulopathy by driving the STAT3/TF axis.

## Discussion

Viral pneumonia-associated coagulopathy remains a critical determinant of mortality in diseases ranging from influenza to COVID-19 (1, 12, 40, 41). However, the specific molecular drivers that initiate the coagulation cascade in the infected lung remain incompletely understood. In this study, we identify NMI as a pivotal DAMP that promotes this pathological process. Through integrated transcriptomic analyses and *in vivo* models, we show that NMI is robustly upregulated in viral pneumonia and strongly correlates with coagulation signatures. Bioinformatic analyses revealed that NMI expression is intrinsically coupled with alveolar epithelial hyperactivation. Mechanistically, we delineate a novel epithelial autocrine loop: secreted NMI engages the STAT3 signaling axis on alveolar epithelial cells to drive the transcriptional upregulation of TF, rapidly precipitating the extrinsic coagulation cascade. *In vivo*, disrupting this axis via genetic ablation of *Nmi* or pharmacological inhibition of STAT3 effectively mitigates lung pathology and prevents microthrombus formation, highlighting the therapeutic potential of the NMI-STAT3-TF axis.

### Extracellular NMI drives localized coagulopathy via a synergistic paracrine-autocrine signaling network.

While NMI has canonically been characterized as an intracellular regulator, recent evidence underscores its emerging role as an extracellular DAMP. Previous studies primarily positioned NMI as a pro-inflammatory mediator, promoting macrophage activation via NF-κB pathways in sepsis and multiple sclerosis (21, 22). However, our study unveils a fundamentally distinct mechanism in the context of viral pneumonia: extracellular NMI functions as an important contributor to localized pro-coagulant activation. As supported by our single-cell profiling, infiltrating myeloid cells, particularly macrophages and neutrophils, serve as a major reservoir of NMI and are poised to exert potent paracrine stimulatory effects on the neighboring lung tissue. However, our data demonstrate that the alveolar epithelium is not merely a target of these immune-derived signals. Rather, it acts as an active participant in the thrombo-inflammatory cascade. The local induction of TF is likely fueled by a synergistic dual mechanism: paracrine NMI secreted by macrophages (21, 22), critically compounded by an NMI-driven autocrine loop autonomously maintained by the hyperactivated epithelium itself. This integrated “paracrine-autocrine” signaling network structurally amplifies the extracellular DAMP pool, thereby sustaining the localized pro-coagulant microenvironment and accelerating thrombosis.

### Potential mechanisms of extracellular NMI release.

While our study firmly supports the extracellular accumulation and pathogenic activity of NMI, the precise molecular mechanisms governing its release during viral pneumonia remain incompletely defined. Our data demonstrate that extracellular NMI is significantly increased under virus-mimicking inflammatory conditions; specifically, poly(I:C) stimulation induces NMI release from alveolar epithelial cells, and our scRNA-seq analysis reveals broad upregulation of *Nmi* across multiple pulmonary cell populations in infected lungs. These findings suggest that extracellular NMI originates from more than one cellular source, primarily activated alveolar epithelial cells and infiltrating immune cells. In line with this, previous studies have established that NMI can function as a pro-inflammatory DAMP and is actively released by macrophages during infection- or injury-associated inflammatory responses (21, 22). Given that DAMPs can enter the extracellular space through multiple routes, we postulate that NMI accumulation in severe viral pneumonia involves a combination of active and passive processes. The intense inflammatory microenvironment and profound epithelial injury likely promote the passive release of NMI from damaged or dying cells following membrane disruption. Concurrently, activated macrophages and hyperactivated epithelial cells may robustly contribute to active NMI secretion. Future investigations are required to definitively elucidate whether NMI release depends on the classical ER–Golgi pathway, unconventional secretion mechanisms, extracellular vesicle-mediated transport, or secondary passive release from necrotic cells.

### STAT3 signaling mediates NMI-dependent TF upregulation.

Mechanistically, the identification of the STAT3-TF axis as the downstream effector of NMI highlights a unique pro-coagulant pathway. Unlike classical DAMPs such as HMGB1, which primarily induce TF via the TLR4-NF-κB axis (17, 42), our single-cell GSEA profiling specifically identified the JAK-STAT signaling signature as the top-ranked cascade correlated with coagulation in *Nmi*-high alveolar epithelial cells. Consistent with this statistical dominance, pharmacological inhibition of STAT3 (Stattic) significantly suppressed TF expression *in vitro*. Furthermore, since STAT3 has broad inflammatory functions, the reduction of TF expression observed *in vivo* following Stattic treatment likely reflects a synergistic dual mechanism: a direct, cell-autonomous suppression of STAT3-driven TF transcription within the epithelium, combined with a secondary effect from attenuated systemic inflammation that removes major paracrine stimuli for TF induction. Nevertheless, this suppression was not absolute, indicating that STAT3 is not the sole relevant mediator and that pathological TF induction is orchestrally regulated by multiple signaling cascades. Indeed, our multi-omics analyses also flagged alternative stress-responsive networks, including the TLR, NF-κB, PI3K-Akt, and MAPK pathways. Rather than functioning in isolation, these cascades likely cooperate synergistically to sustain the thrombo-inflammatory microenvironment. We postulate a highly plausible crosstalk mechanism: extracellular NMI acting as a DAMP may simultaneously activate the NF-κB, PI3K-Akt, and MAPK axes in infiltrating myeloid cells (such as macrophages), triggering the robust secretion of pro-inflammatory cytokines like IL-6 and TNF-α (21, 43, 44). Crucially, these myeloid-derived cytokines, particularly IL-6, can serve as potent upstream paracrine activators of epithelial STAT3 signaling, thereby indirectly accelerating TF expression and fueling a vicious positive feedback loop between inflammation and coagulation. Future studies are warranted to further dissect the synergistic crosstalk between these parallel pathways in DAMP-mediated thrombosis.

### Targeting upstream NMI signaling as a therapeutic strategy for viral coagulopathy.

The therapeutic implications of targeting the NMI axis are particularly significant given the current challenges in managing viral coagulopathy. TF serves as the critical trigger for coagulation, and its uncontrolled exposure precipitates an escalating cycle of tissue injury and microvascular thrombosis (45, 46). While inhibiting the TF-FVIIa complex or utilizing direct anticoagulants has shown efficacy in animal models (47), these strategies have consistently failed to demonstrate robust clinical benefit in human trials, largely due to severe bleeding risks (48, 49). Consequently, identifying an upstream target that decouples pathological thrombosis from physiological hemostasis is imperative. Our *in vivo* poly(I:C)-induced viral pneumonia model establishes NMI as such a target. We demonstrate that NMI inhibition effectively dampens the hypercoagulable state, evidenced by diminished TF expression, reduced fibrin deposition, and lower systemic levels of TAT complexes and PAI-1. By inhibiting the upstream DAMP rather than downstream clotting factors, targeting NMI alleviates lung pathology while potentially preserving baseline hemostatic function.

### Association of circulating NMI with disease severity and prognosis.

Furthermore, circulating NMI holds promise as a potential biomarker. Recent evidence highlights NMI as a novel prognostic biomarker for CAP, outperforming conventional markers in predicting 30-day mortality (25), while its homolog, IFP35, strongly correlates with COVID-19 severity (50). Aligning with these observations, our current study confirms a robust systemic elevation of NMI in both COVID-19 and influenza patients, demonstrating promising potential in distinguishing infected individuals from healthy controls. Consequently, assessing NMI levels could facilitate early risk stratification for thrombotic complications in viral pneumonia, providing a rational foundation for precision interventions.

### Limitations and future work.

Despite these insights, our study has limitations. First, the ROC analyses performed in this study rely on relatively limited, retrospective transcriptomic datasets. The diagnostic and prognostic potential of circulating NMI must be rigorously validated in independent, large-scale prospective clinical cohorts before it can be considered for clinical application. Second, while NMI is known to signal via the TLR4 receptor in macrophages (21, 22), the specific cell surface receptor through which NMI activates the STAT3 pathway in epithelial cells remains to be definitively identified. Future CRISPR-screening studies are warranted to pinpoint this epithelial NMI receptor. Third, we utilized a poly(I:C)-induced model to standardize the epithelial injury and pro-coagulant response; validation in live influenza or SARS-CoV-2 infection models will be an important next step to confirm broad translational relevance.

## Conclusions

We define a novel epithelial autocrine “NMI-STAT3-TF” axis that contributes to poly(I:C)-induced pulmonary coagulopathy and may be relevant to viral pneumonia-associated thrombo-inflammatory responses. Our findings provide a compelling mechanistic rationale for targeting this pathogenic loop, specifically through NMI or STAT3 inhibition, as a promising therapeutic strategy to mitigate life-threatening thrombotic complications in severe viral pneumonia.

## Data Availability

The original contributions presented in the study are included in the article/**Supplementary Material**. Further inquiries can be directed to the corresponding authors.
