# Supplementary material for: N-myc and STAT interactor promotes poly(I:C)-induced pulmonary coagulopathy via STAT3-dependent tissue factor
Source: Front Immunol. 2026 Jul 2;17:1873798. doi: 10.3389/fimmu.2026.1873798 (PMC13372619; doi:10.3389/fimmu.2026.1873798)
Supplement: Supplementary file 1 [file DataSheet1.pdf]

Supplementary materials

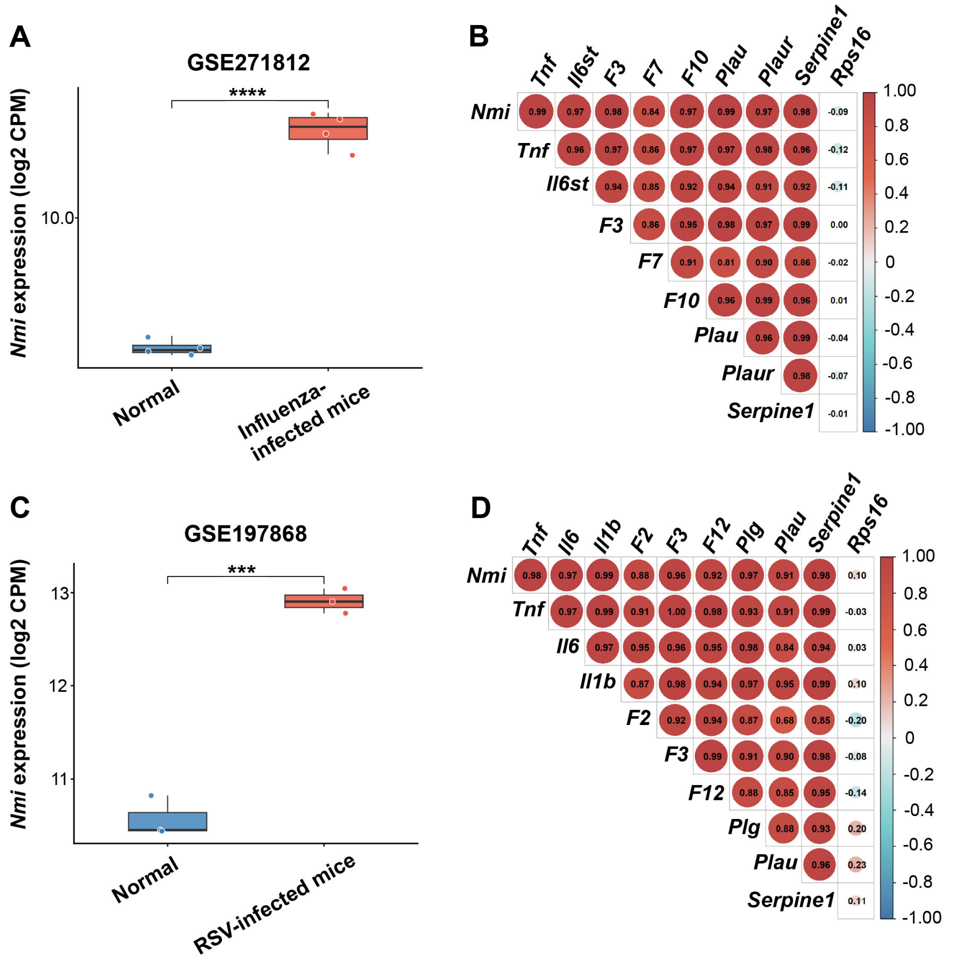

Supplementary Figure S1. *Nmi* is significantly upregulated in murine viral pneumonia models and correlates with pro-inflammatory and coagulation mediators. (A) Expression levels of *Nmi* in lung tissues from H1N1 influenza-infected mice compared to the control group (GSE271812). (B) Correlation analysis between *Nmi* expression and key pro-inflammatory cytokines, coagulation factors, and the housekeeping gene *Rps16* in the H1N1 cohort. (C) *Nmi* expression in lung tissues from RSV-infected mice versus control mice (GSE197868). (D) Correlation analysis between *Nmi* expression and pro-inflammatory cytokines, coagulation factors, and the housekeeping gene *Rps16* in the RSV cohort. Statistical significance was determined using an unpaired two-tailed Student's *t*-test. ***$p < 0.001$, ****$p < 0.0001$. Correlations were assessed using Pearson correlation coefficients.

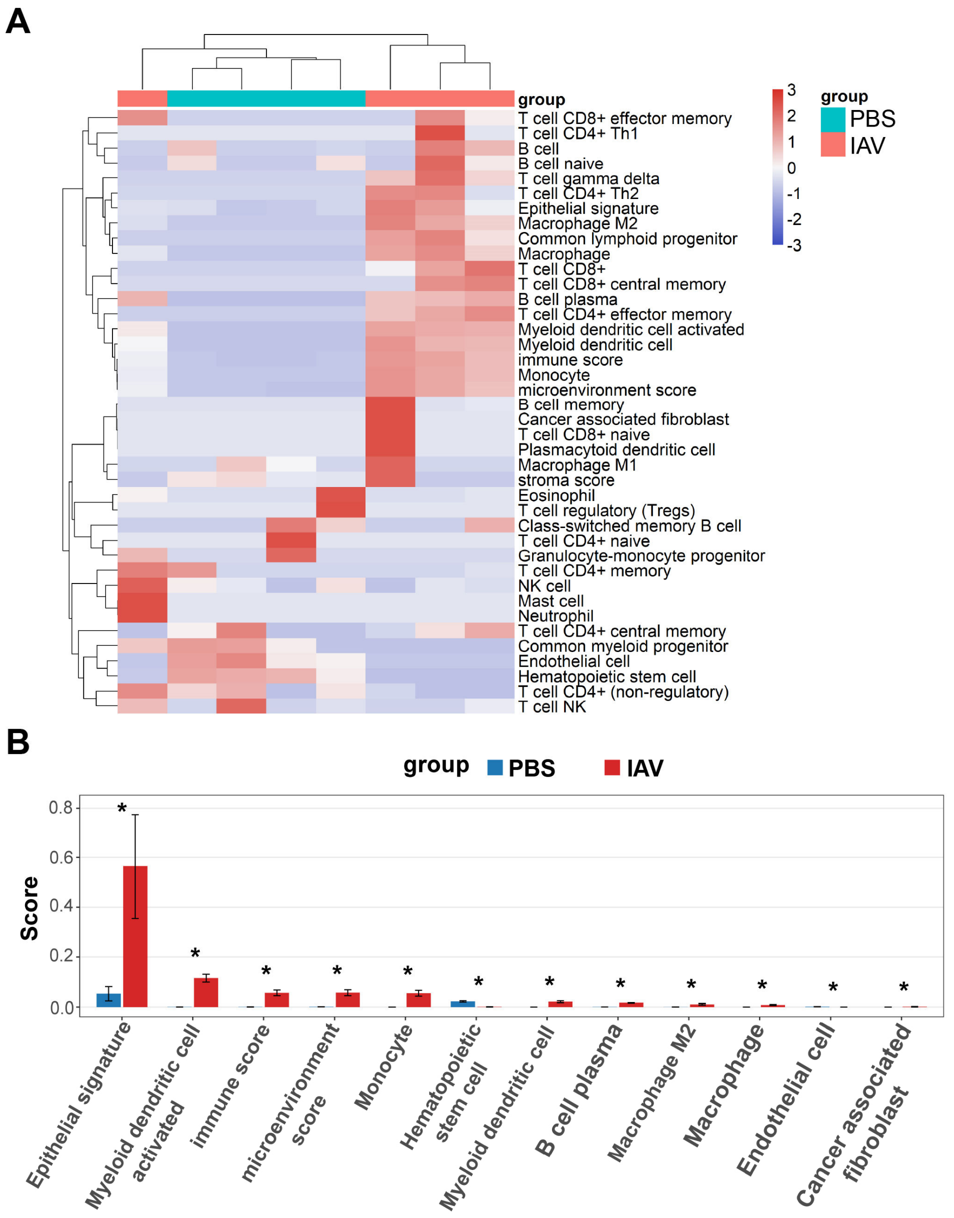

Supplementary Figure S2. Remodeling of the lung cellular landscape in influenza pneumonia. (A) Heatmap illustrating the infiltration profiles of 40 distinct cell types (inferred via ssGSEA) in lung tissue samples from influenza-infected mice (IAV) compared to PBS controls (GSE271812). (B) Comparative abundance of key immune and stromal cell populations between the PBS and IAV groups. Statistical significance was determined by the Wilcoxon rank-sum test. *$p < 0.05$.

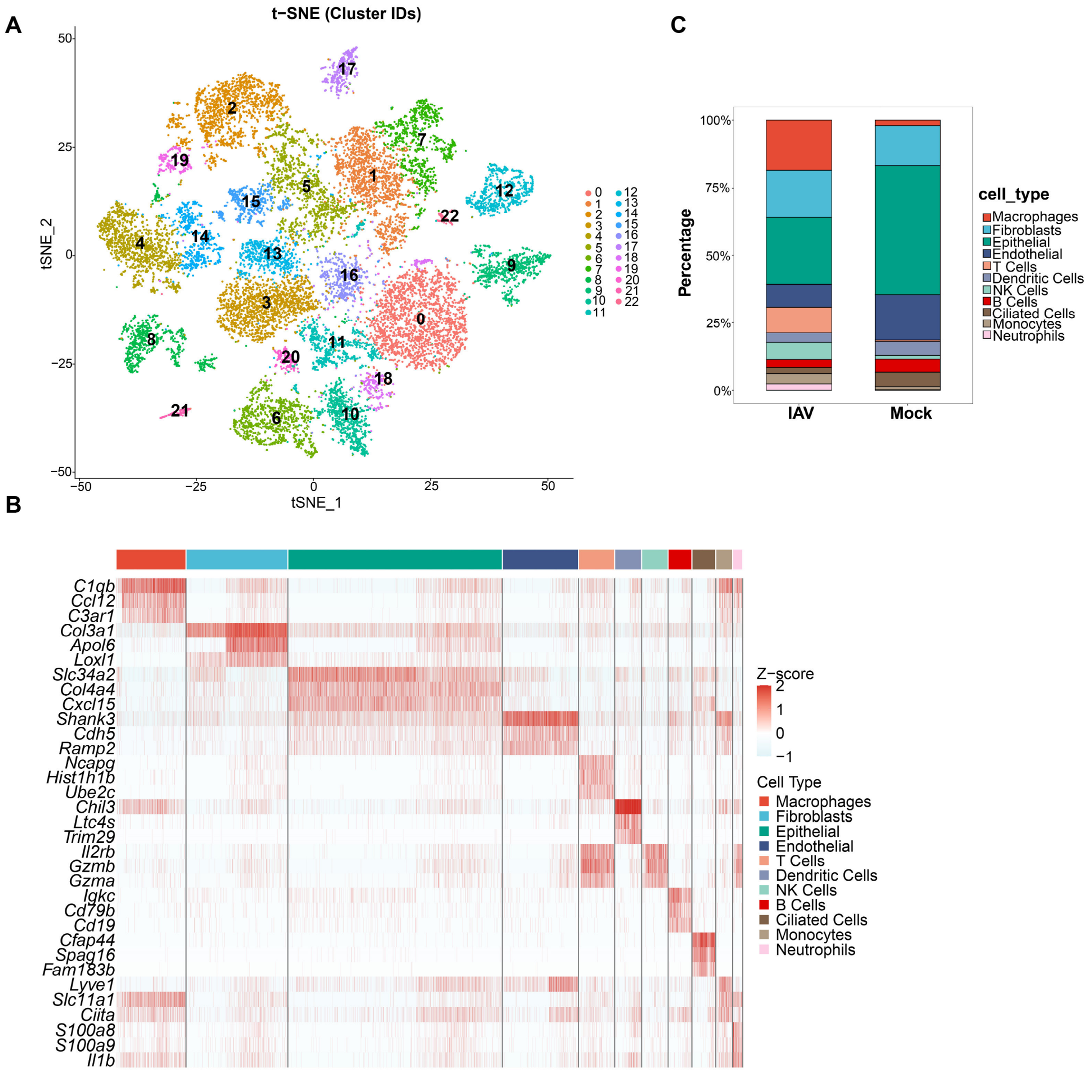

Supplementary Figure S3. Detailed characterization and annotation of single-cell clusters. (A) t-SNE plot showing all 23 clusters colored by cluster ID (0–22). (B) Heatmap displaying the top 3 marker genes for each annotated cell type. The color scale represents the Z-score of gene expression. (C) Bar plot illustrating the relative proportions of major immune and stromal cell lineages between Mock and IAV groups, highlighting differences in cellular composition.

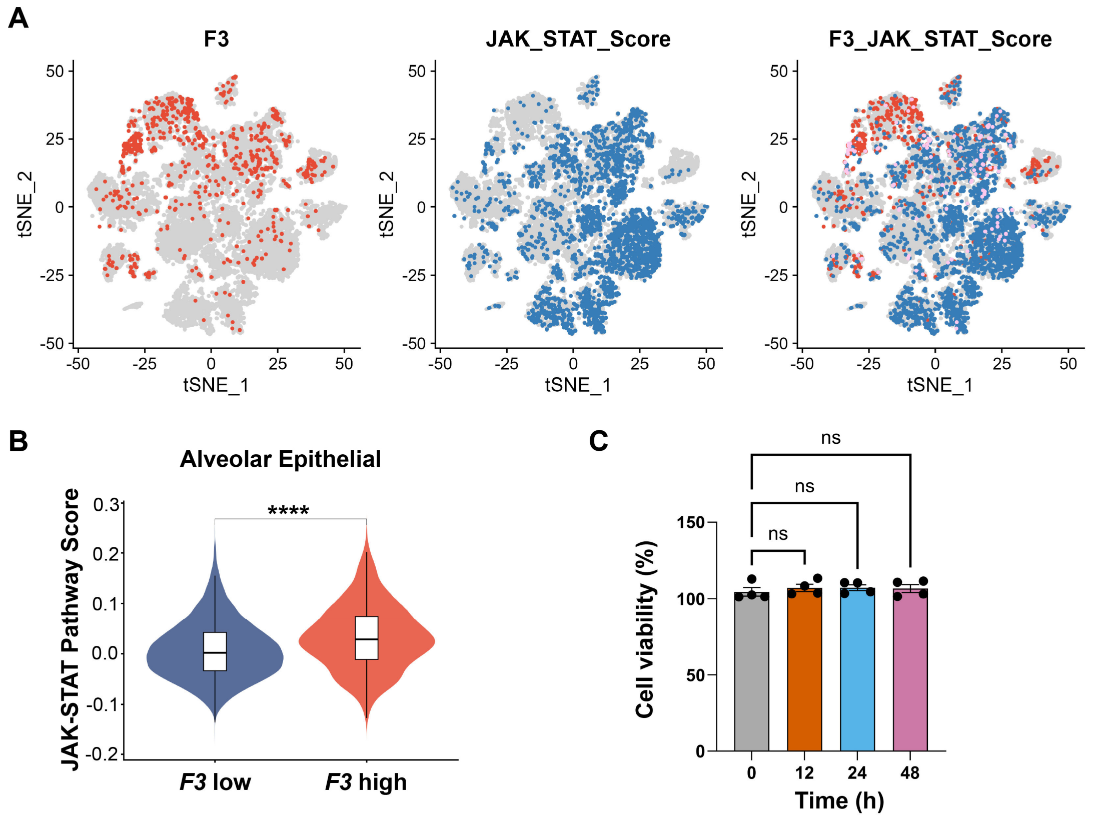

Supplementary Figure S4. Activation of the JAK-STAT pathway characterizes *F3*-high epithelial cells during viral infection. (A) Dual-feature visualization highlighting the spatial overlap between the JAK-STAT signaling score and *F3* expression across all annotated lung cell clusters. (B) Violin plot comparing the JAK-STAT signaling score between *F3*-high and *F3*-low epithelial subpopulations. Data indicate a significant upregulation of JAK-STAT activity in *F3*-expressing cells. Statistical significance was determined using the Wilcoxon rank-sum test. (C) A549 cells were incubated with Stattic (100 nM) for 0, 12, 24, and 48 hours at 37°C, followed by cell viability assessment using the CCK-8 assay. Data are presented as mean ± SEM. Statistical significance was determined using one-way ANOVA. ns, not significant, ****$p < 0.0001$.

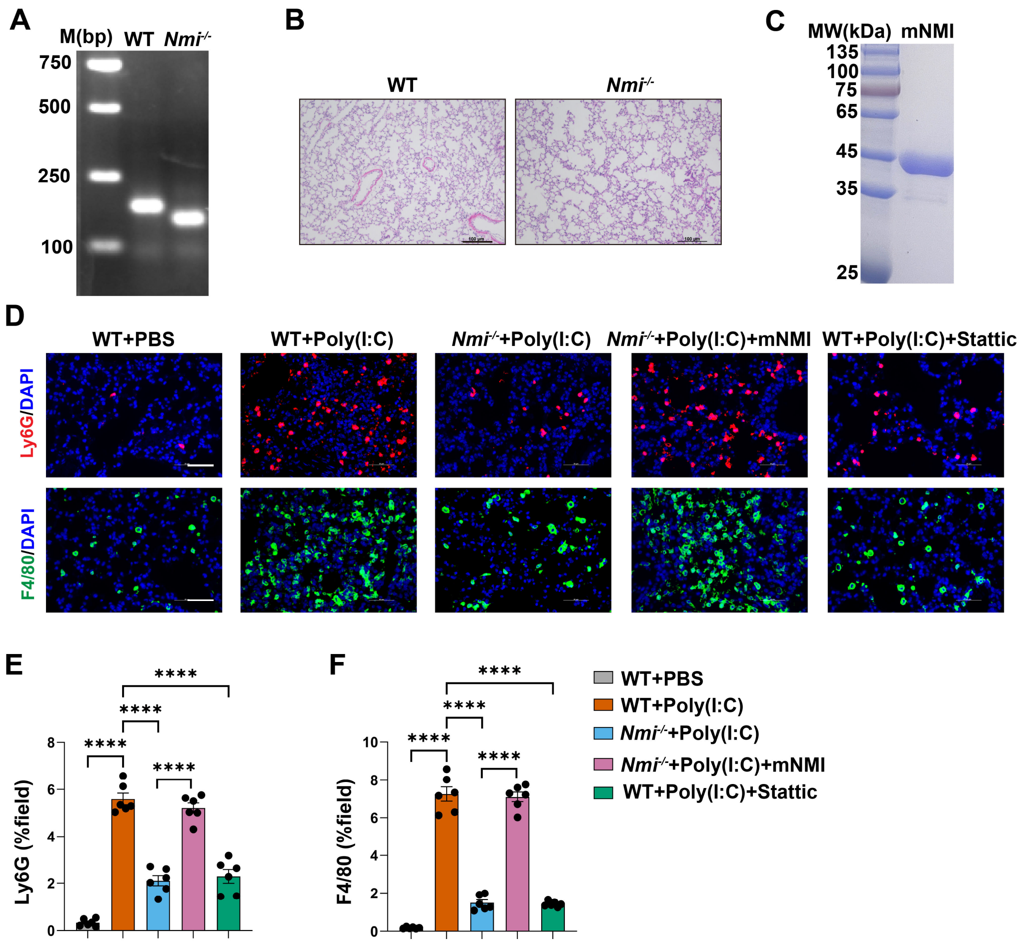

Supplementary Figure S5. Model validation and NMI-STAT3-mediated pulmonary immune infiltration. (A) PCR genotyping of WT and *Nmi*$^{-/-}$ mice. (B) Representative H&E-stained lung sections from naive WT and *Nmi*$^{-/-}$ mice, demonstrating that *Nmi* deletion does not induce spontaneous morphological abnormalities in the lung tissue. Scale bar, 100 μm. (C) SDS-PAGE and Coomassie Brilliant Blue staining of mNMI. mNMI protein was resolved to confirm a purity of >95%. (D) Representative immunofluorescence staining of lung sections from the indicated groups. Tissues were labeled with antibodies against Ly6G (neutrophils) and F4/80 (macrophages). Scale bar, 25 μm. Quantitative analysis of the percentage of area occupied by (E) Ly6G-positive neutrophils and (F) F4/80-positive macrophages in lung sections. Each dot represents an individual mouse. Data are presented as mean ± SEM. Statistical significance was determined using one-way ANOVA. ****$p < 0.0001$.
